# Structural Operativity Evaluation of Strategic Buildings through Finite Element (FE) Models Validated by Operational Modal Analysis (OMA)

**DOI:** 10.3390/s20113252

**Published:** 2020-06-07

**Authors:** Dora Foti, Nicola Ivan Giannoccaro, Vitantonio Vacca, Michela Lerna

**Affiliations:** 1 Dipartimento di Scienze dell’Ingegneria Civile e dell’Architettura, Politecnico di Bari, 70126 Bari, Italy; michela.lerna@poliba.it; 2 Dipartimento di Ingegneria dell’Innovazione, Università del Salento, 73100 Lecce, Italy; 3 Consiglio Nazionale delle Ricerche–Istituto di Geologia Ambientale e Geoingegneria, 00015 Roma, Italy; vitantonio.vacca@cnr.igag.it

**Keywords:** nondestructive techniques, operational modal analysis, ambient vibrations, FE model, structural operativity

## Abstract

In this paper, a non-destructive technique based on the monitoring of the environmental vibrations of two strategic buildings by positioning accelerometers in well-defined points was used for fixing their dynamic behavior. The accelerometers measurements were elaborated through Operational Modal Analysis (OMA) techniques, in order to identify natural frequencies, damping coefficients, and modal shapes of the structure. Once these parameters have been determined, a numerical model calibrated on the identified frequencies and verified on the corresponding mode shapes was created for each building. The structural operational efficiency index of the buildings was determined by using the Seismic Model Ambient Vibration (SMAV) methodology, which allows us to evaluate their seismic vulnerability. The results obtained from the experimental analysis (on three different tests for each analyzed building) concern the frequencies and the modal shapes of the structure. They have been compared to the results of the finite element model, with a very small error, indicating a good quality of the analysis and also the possibility of using directly well-tuned models for verifying the structural operating indices.

## 1. Introduction

In this paper, we introduce a procedure that can be a helpful tool to trace the health condition of strategic buildings through Finite Element (FE) models validated by means of a nondestructive technique for the dynamic analysis.

In recent years, among nondestructive techniques used for performing structural building analyses and evaluating their operational status, Operational Modal Analysis (OMA) has become a useful technique for obtaining the effective state of safety and health of a structure. 

As with all the techniques, OMA is useful for modal parameter identification from output only analyses [1,2,3,4,5,6,7,8,9,10]. The knowledge of the modal characteristics of structures, in fact, becomes essential for the model analysis and validation; it can guarantee the safety and practicability of the structure even in the case of unpredictable and high-energy stress events such as earthquakes. 

Recent research has focused on the effects of damage induced by past earthquakes and the effects of masonry infills [11,12,13].

A similar validation is proposed in [14] for the case of bridges. A comparison is proposed of the measured and numerical dynamic responses of two footbridges in order to define the performance assessment to spatial variation of earthquake ground motion.

The reliability of the vulnerability assessment of structures is an essential prerequisite for the assessment of seismic loss, territorial management, and risk mitigation. In the built heritage, all the structures with considerable strategic importance then assigned to accommodate essential activities to the community (i.e., fire stations, hospitals, town halls, offices open to the public) play a very important role. Therefore, the assessment of vulnerability of strategic buildings has become a crucial point for planning risk mitigation.

In this context, OMA can be considered an effective non-destructive tool for performing accurate structural analyses and assessing the actual operational status of strategic and relevant structures [15]. The analysis of the response to environmental vibrations, in fact, translates into a valid method for the dynamic identification of strategic role structures. OMA can contribute to understanding the behavior in the presence of high-energy dynamic and environmental excitations and to develop a numerical model to estimate the structural response in weak excitation [16,17].

In recent years, numerous studies have proved that OMA is an adequate monitoring methodology to provide reliable predictions for assessing the vulnerability of reinforced concrete structures [18,19,20,21,22,23]. In particular, environmental vibration tests have become the main experimental method available for evaluating the dynamic behavior of structures on a large scale because the excitation equipment is not necessary. In this way, it involves a minimum interference with the ordinary use of the structure. This aspect makes it possible to monitor in situ and dynamically identify strategic structures. In order to fulfill their strategic role, in fact, these buildings must remain fully operational even during the ambient vibration testing [24]. 

In [25], a method is proposed for assessing the vulnerability of buildings that remain operational after strong dynamic excitement. The method is based on the identification of experimental modal parameters from environmental vibration measurements; then a linear spectral analysis computing the maximum structural drifts of the building caused by an assigned external force is performed. The operating conditions are then assessed by comparing the maximum drifts of the buildings with the reference value assigned by the Italian technical code for the Operational Limit State [15]. The operational index and the operational probability curve define the vulnerability of a building analyzed under strong dynamic excitement.

In [26], a Seismic Model from Ambient Vibrations (SMAV) is extensively described. It is useful for the capacity assessment of strategic buildings. The approach can be an effective method to establish a ranking of buildings’ vulnerability. It is then possible to select the structures for emergency management or to know how to distribute the economic resources for their repair and retrofitting. 

In order to apply a structural safety assessment approach, the in-situ dynamic tests are performed to validate the numerical model. In particular, the experimental data obtained through OMA can be used for the updating process of accurate Finite Element (FE) models in order to estimate their structural properties. The main purpose of the model updating procedure is to minimize the differences between the numerical and experimental modal parameters (frequencies and modal forms) bringing the numerical model closer to the experimental one [27]. In the last decade, the process of FE model updating to establish the dynamic characteristics of a system from the experimental model has been applied to different construction typologies [28,29,30,31]. 

In the present work, the dynamic identification of two strategic role buildings carried out via OMA is described. In particular, the data of the ambient vibrations on the Provincial Command of Fire Fighters building in Castellaneta (Taranto, Italy) and on the City Hall of Ginosa (Taranto, Italy) have been recorded by means of accelerometers. They are two structures very different from one another both in terms of the shape and the materials utilized. The information obtained from the in-situ monitoring has been analyzed and processed to reach the mean modal parameters (modal shapes) of these two strategic buildings. The results of the tests have been used for calibrating the FE models of the buildings that have been appropriately tuned to the first three identified frequencies in order to understand how reliable the numerical models are in predicting the dynamic behavior of structures. Through the SMAV procedure, the consistency between the experimental operational indices and the numerical operational ones was evaluated. The results predicted by a SMAV experimental analysis have been successfully compared with the results obtained by calculating the numerical operational indices directly on the tuned models. The success of the proposed procedure is important because the experimental effort for tuning FE models is much lower than for experimentally estimating the mode shapes. 

The procedure described in this paper can be a helpful tool to trace a health condition mapping of strategic buildings in a defined area through validated FE models to dynamic behavior prediction.

## 2. Cases Studies

The buildings under study are classified as having a “strategic interest”, following the Italian Technical Code [15]; they belong to those buildings whose uses during the seismic events are fundamental for the aims of the Civil Protection Department (use class IV). The area where the two buildings are located, in terms of seismic hazard has been classified as zone 3, following the regional council resolution 2/3/04 n.153. In zone 3, strong earthquakes are less probable than in zones 1 and 2. A base hazard is assigned to this zone with a probability of exceedance equal to 10% in 50 years, that is, in terms of peak acceleration on stiff soil (a_g_) equal to 0.05 < a_g_ < 0.15. It corresponds to an anchoring horizontal acceleration of the elastic response spectrum equal to 0.15 (a_g_/g).

### 2.1. Structure A: Provincial Command of Fire Brigade Building in Castellaneta (Taranto, Italy)

**Figure 1:**
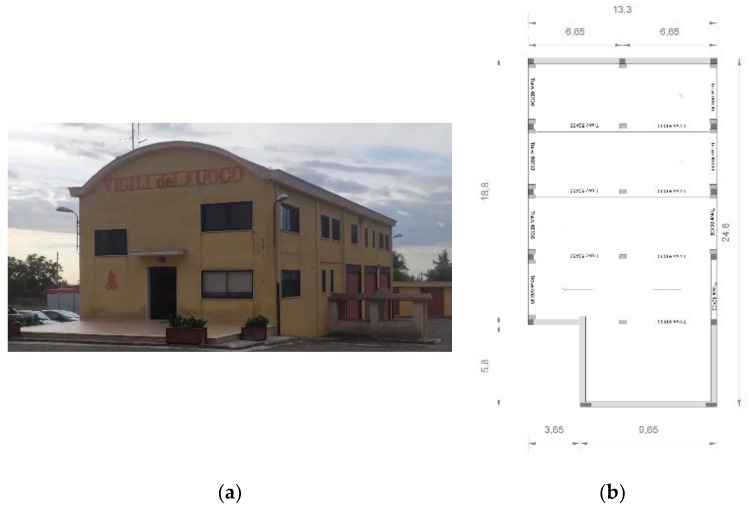
(**a**) Frontal view of the building; (**b**) Plant view of Structure A.

The Provincial Command of Fire Brigade building in Castellaneta (Taranto, Italy) is located in the southwest suburbs of the town. It can be reached by SS 7 road that connects Castellaneta to Palagiano (Taranto). The building has two floors above the ground and consists of a frame structure in reinforced concrete and load-bearing masonry in tuff blocks. The two levels above the ground have different heights: the ground floor, hosting the garage of the Fire Brigade vehicles and the related workshop, has a height of about 4.5 m. The upper floor, hosting the offices, the operations center and the rooms, has a variable height, since the roof is a barrel vault in reinforced concrete with a height of 2.47 m (Figure 1). An intermediate floor is present at a height of 4.5 m. At present, the structure is a "mixed" type one: most of the loads are supported by the reinforced concrete frames and walls of the basement; these structures have additional supports by the load-bearing walls made of 40 cm thick tuff blocks. The floors are made up in brick-cement having a total thickness of 25 + 5 cm. The roof of the entire building consists of a lowered arch in reinforced concrete. The various floors are vertically connected by reinforced concrete stairs of the rampant type. The foundations essentially consist of connected plinths in reinforced concrete.

### 2.2. Structure B: City Hall of Ginosa (Taranto, Italy)

The City Hall of Ginosa is a building located in the northeast part of the town close to the historical center. 

**Figure 2:**
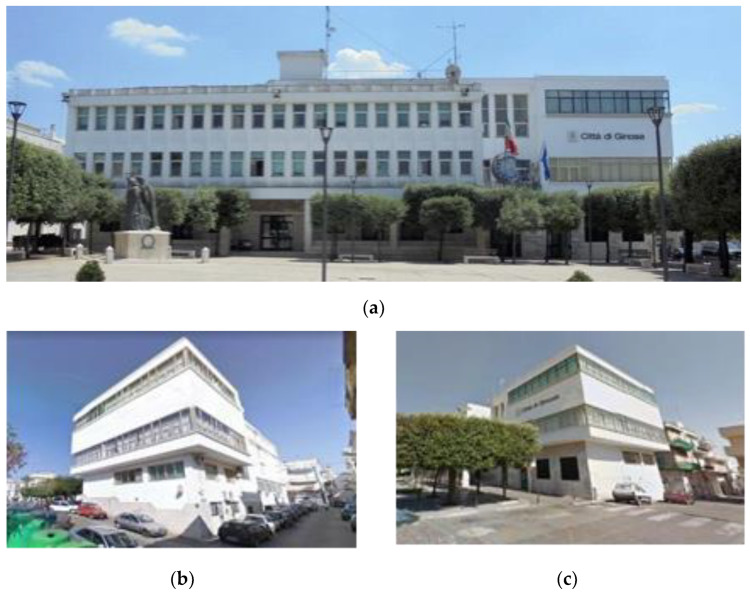
(**a**) North view of the City Hall building; (**b**) northwest view of the City Hall building; (**c**) southwest view of the City Hall building—Structure B.

It was built in the 1970s and is characterized by a framed structure in reinforced concrete (Figure 2) on five level: (5.93 m, 9.27 m, 12.61 m, 14.41 m, and 15.09 m).

**Figure 3:**
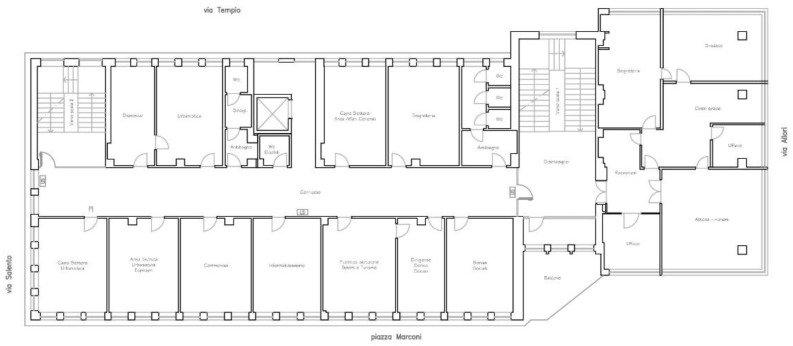
Plant type view of Structure B.

Specifically, the first two levels constitute the first and second floors are used as offices, on the third level there is an archive with a solar roof accessible for maintenance only, while the fourth and fifth levels consist of solar roofs accessible for maintenance only. The overall plan dimensions are approximately 45 × 19 m (Figure 3). The masonry infills are made of 40 cm thick tuff blocks and the floors are made of reinforced concrete and hollow tiles having a total thickness of 25 + 5 cm. 

## 3. Environmental Vibration Testing

The vibration measurement tests were carried out to characterize the modal properties of both buildings. Regarding the monitoring system: the chain of acquisition was composed of high sensitivity seismic accelerometers ICP PCB 393B31 (sensitivity = 10 V/g) monoaxial piezometric type, with a frequency range from 0.1 to 200 Hertz, a multi-channel acquisition system (National Instruments (NI)—NI 9230), with three simultaneously sampled analog inputs with a ±30 V input range, and a platform NI Compact DAQ DSA with eight slots. In each one, it is possible to integrate a NI 9234 chassis able to acquire simultaneously from the channels. The accelerometers enable us to obtain accelerometers data for low frequency and low acceleration values. They have been installed through a threaded pin on a cubic-shaped metal element in order to ensure the orthogonality of the couple of accelerometers placed on the same position. The cubic support element is then fixed to the structure. All components were connected by co-axial cables with low impedance and with a length of 15 m at a portable compact platform to data collecting. During both test campaigns for Structure A and B, the structural response was recorded by capturing absolute accelerations at different significant points with a sampling frequency of 512 Hz, for a total duration of 10 minutes for each test. Three tests were carried out for each building in order to ensure the repeatability of the identification results; hereafter, they will be named Test 1, Test 2, and Test 3 in chronological order for each structure. The management of the acquisition and archiving of the data was carried out by means of a piece of software developed in Lab View [32].

The location of the accelerometers was planned based on the specific structural configurations, in the *x* and *y* axis directions. For each building, three different tests have been analyzed with two OMA techniques [33], one in the frequency domain, the Enhanced Frequency Domain Decomposition (EFDD) method, and the second in the time domain, the Crystal-Clear Stochastic Subspace Identification (CC–SSI) method. The repeatability of the identified frequency values with the two techniques, for all the considered tests, allowed us to be very confident about the identified frequencies and the mode shapes for both structures. 

### 3.1. Structure A

**Figure 4:**
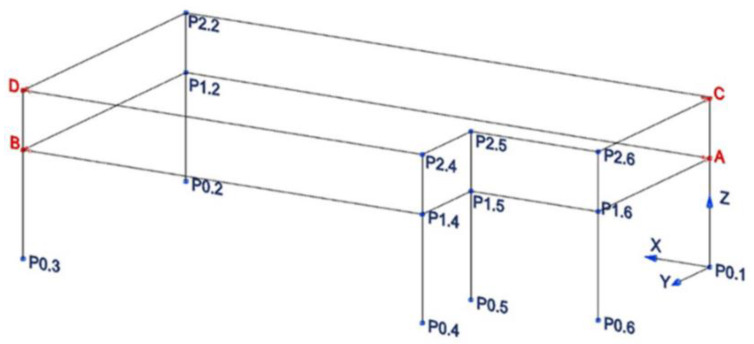
Identified monitoring points—Structure A.

**Figure 5:**
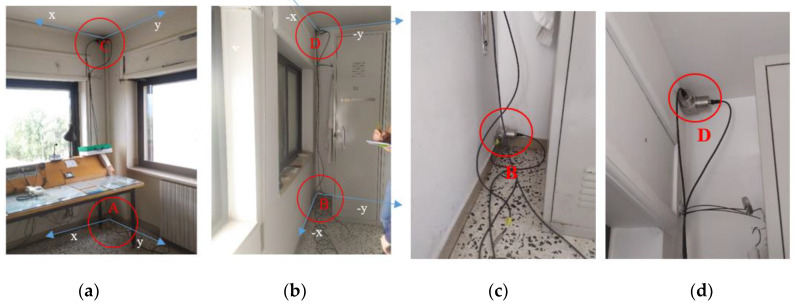
(**a**) Acquisition points A and C and the adopted reference system; (**b**) acquisition points B and D; (**c**) details of accelerometers positioned in point B by means of the cubic element; (**d**) details of accelerometers mounted on point C by means of the cubic element.

In the case of Structure A, with a simple and schematic configuration, only eight accelerometers were positioned, four for each floor (floor 1 at 4.50 m and floor 2 at 6.97 m). A more detailed scheme about the accelerometers positioning at different levels of Structure A is shown in Figure 4. In each measurement point (red points in Figure 4), two accelerometers have been positioned along *x* and *y* direction, respectively. Figure 5 shows the four monitoring points and the adopted reference system. 

A preliminary analysis of the recorded data has allowed us to consider all the accelerometers properly functioning without anomalies; therefore, all the accelerometers data were considered for the modal identification by using modal techniques. 

#### OMA for Structure A

**Figure 6:**
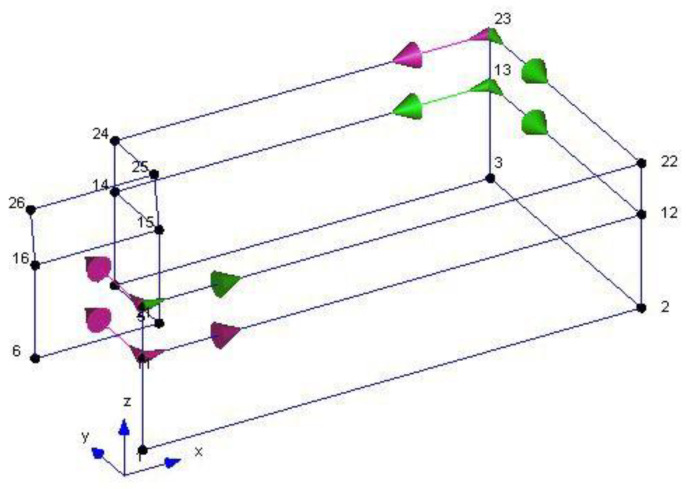
Geometry of Structure A for Operational Modal Analysis (OMA) along with accelerometers positions and directions.

**Figure 7:**
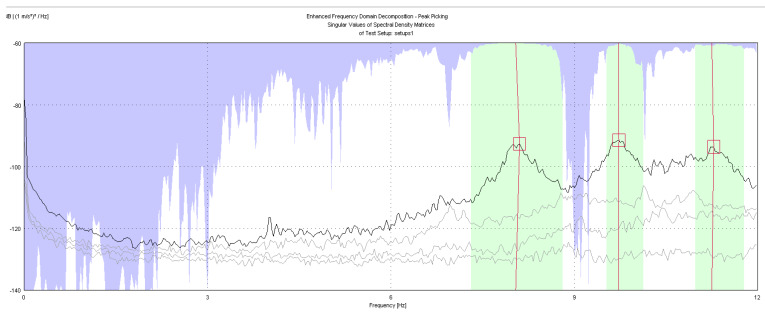
OMA identification with Enhanced Frequency Domain Decomposition (EFDD) method, Test 1.

**Figure 8:**
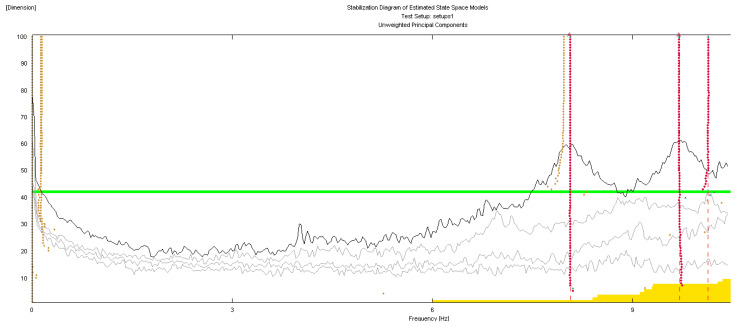
OMA identification with Stochastic Subspace Identification (SSI) method, Test 1.

**Figure 9:**
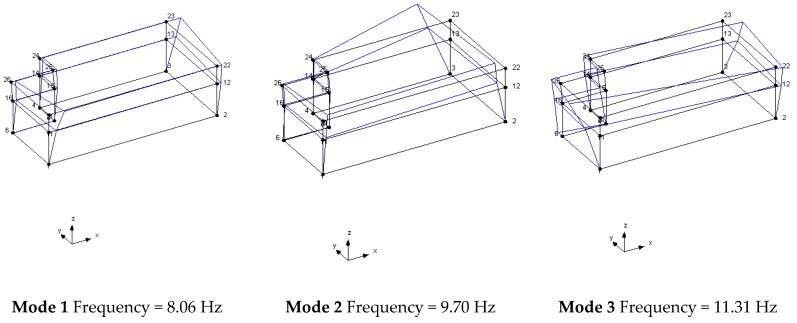
Mode shapes identified using SSI method – Structure A, Test 1.

The geometry of the structure has been reconstructed for the identification phase with 18 points having the same nomenclature as in Figure 4. The building geometry used for the analysis together with the positions and directions of the accelerometers (indicated as arrows) and the *xyz* reference system is shown in Figure 6. The diagrams of EFDD analysis and CC–SSI analysis for Test 1 are shown in Figure 7 and Figure 8. The first three identified frequencies in the range (0–12 Hz) and the description of the corresponding modes for all the analyzed tests (Test 1, Test 2, and Test 3), for both the techniques utilized (SSI in the time domain and EFDD in the frequency domain) are summarized in Table 1. The repeatability of the three identified frequencies on the different tests is evident along with the different techniques ensuring the reliability of the results. In Table 1, the first frequency is related to a flexional mode along *x*-axis, the second frequency to a flexional mode along *y*-axis, and the third frequency to a torsional mode. The identified modes reported in Table 1 are clearly defined from the mode’s animations (Figure 9) and obtained by a linear interpolation on the non-instrumented points. Finally, it is possible to define an average value for the identified values for the first three frequencies of Structure A, resulting equal to 8.05 Hz (standard deviation 0.01), 9.69 Hz (standard deviation 0.09), 11.35 Hz (standard deviation 0.06), respectively. 

### 3.2. Structure B

**Figure 10:**
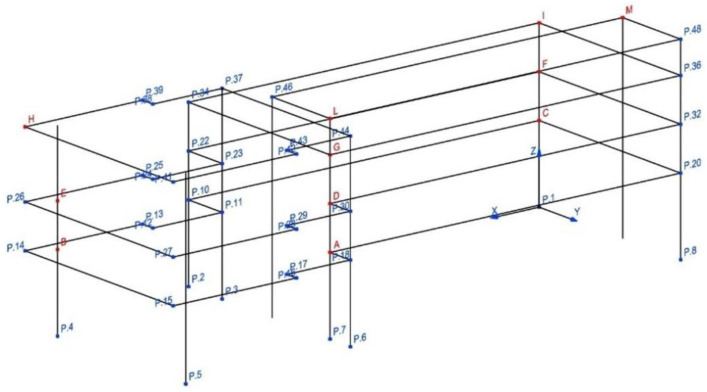
Identified monitoring points—Structure B.

**Figure 11:**
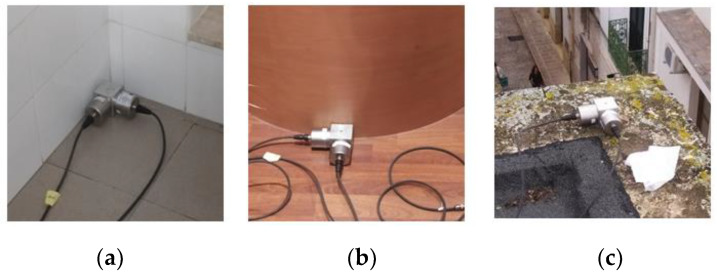
(**a**) Accelerometers in position A; (**b**) accelerometers in position E; (**c**) accelerometers in position M.

Structure B is geometrically and structurally much more complex than Structure A, with several irregularities and asymmetries. After a preliminary analysis, it was decided to use twenty-two accelerometers installed in eleven different points (indicated as letters in Figure 10) for the experimental vibration measurements. Figure 10 shows the 57 points for describing the irregular Structure B and the reference system *x,y* too. In each measurement point (red points in Figure 10), two uniaxial accelerometers orthogonally positioned each other using the same metallic blocks utilized for Structure A have been installed. Figure 11 shows the monitoring points indicated with A, E, and M in Figure 10 and the adopted reference system. 

**Figure 12:**
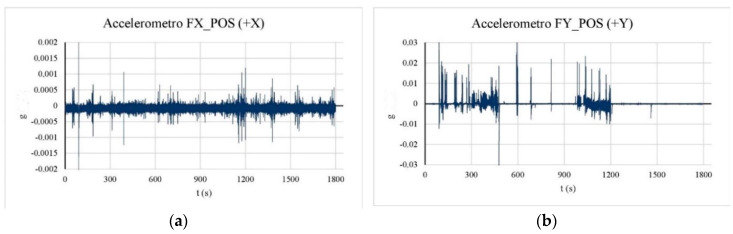
(**a**) Accelerometer time-history in point F, *x* direction; (**b**) accelerometer time-history in point F, *y* direction.

**Figure 13:**
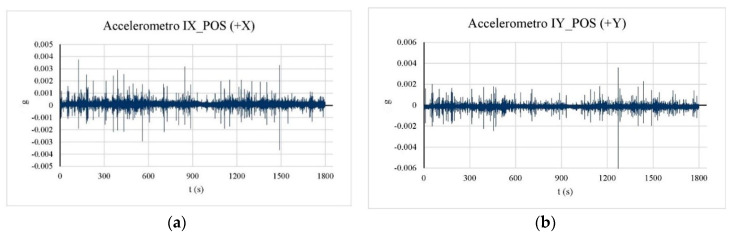
(**a**) Accelerometer time-history in point I, *x* direction; (**b**) accelerometer time-history in point F, *y* direction.

In this case, the preliminary analysis of the acquired time-histories for the three considered tests has enabled us to identify important anomalies for the data carried out from the accelerometers placed in point F (mainly for one of them, third level indicated in Figure 10). For completeness, Figure 12 reports the time histories of the anomalous accelerometer (considering the data of Test 1, Test 2, and Test 3 for a total length of 1800 s) showing important peaks not registered by any other accelerometer. In Figure 13, for comparison purposes, the plot of accelerometers placed in I, in the same position, and at a higher level of position F are shown. For this reason, the accelerometers in position F have not been considered for the identification analysis. 

#### OMA for Structure B

**Figure 14:**
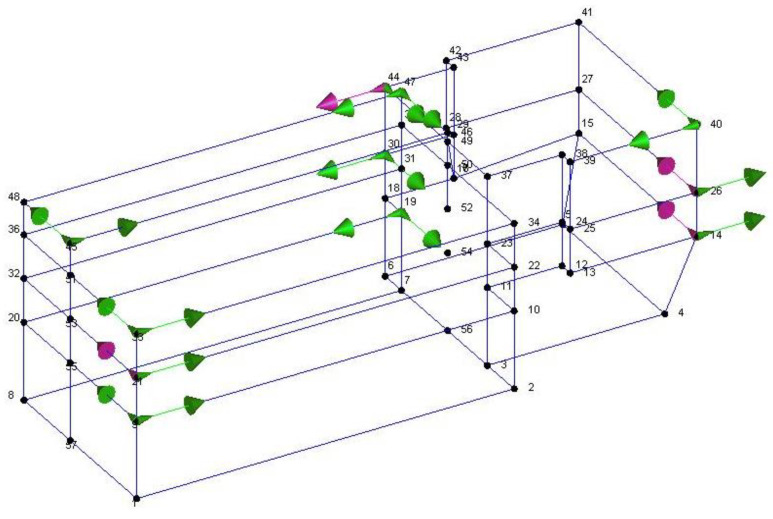
Geometry of Structure B for OMA along with accelerometers positions and directions.

**Figure 15:**
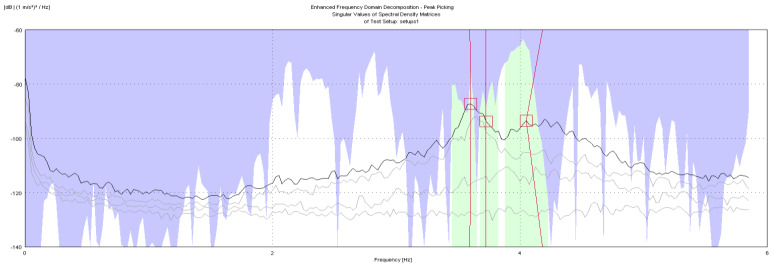
OMA identification with EFDD method, Test 3.

**Figure 16:**
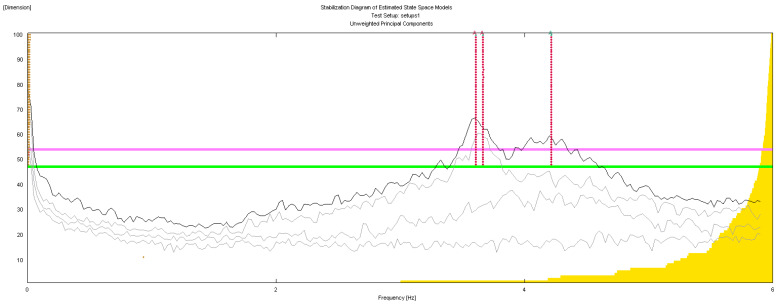
OMA identification with SSI method, Test 3.

**Figure 17:**
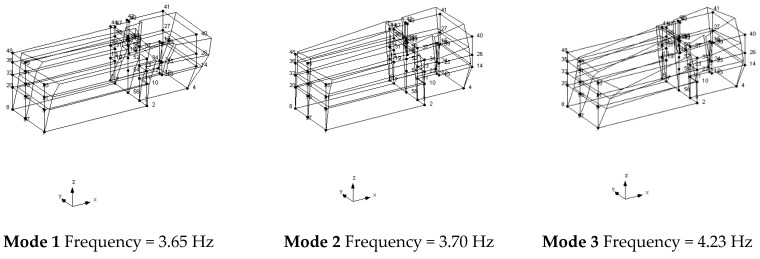
Mode shapes identified with SSI-UPC method—Structure B, Test 1.

The geometric model considered, shown in Figure 14, reports the points, the reference system *xyz* and the accelerometers location and direction (represented as arrows); in correspondence to point F, that is point 23 in the model in Figure 14, no accelerometer has been considered. The diagrams of EFDD analysis and CC–SSI analysis for Test 1 are shown in Figure 15 and Figure 16. The first three identified frequencies in the range [0–6 Hz] and the description of the corresponding modes for all the analyzed test (Test 1, Test 2, and Test 3) and for both techniques (SSI in the time domain and EFDD in the frequency domain) are summarized in Table 2. Moreover, for Structure B, the repeatability of the three identified frequencies on different tests and with different techniques is evident, ensuring the reliability of the results. The very close values of the first two frequencies related to the first flexional modes make it sometimes difficult to distinguish the two values with EFDD technique (see Test 1 and Test 2). In Table 1, the first frequency is related to a flexional mode along *x*-axis, the second frequency to a flexional mode along *y*-axis, and the third frequency to a torsional mode. The characteristics of the identified modes, reported in Table 2, are clearly defined from the mode’s animations, shown in Figure 17, and obtained by means of a linear interpolation on the non-instrumented points.

Finally, it is possible to define an average value as the identified values for the first three frequencies of Structure B equal to 3.62 Hz (standard deviation 0.02), 3.69 Hz (standard deviation 0.03), 4.22 Hz (standard deviation 0.03), respectively.

## 4. Finite Element Models

In this research, FE models of two buildings (Structure A and Structure B) are developed through the model updating process, in order to predict their seismic behavior. The commercial software SAP2000 [34] was employed to create the finite element models. The model-updating phase consisted in the varying and the manual tuning of a set of uncertain modeling parameters, and in the search for the values that ensured the best match with the experimental response. The Finite Element Method (FEM) models of the buildings have been updated by changing the modulus of elasticity, boundary conditions, and mass of the structure. The specific dimensions of structural elements were considered during the updating steps. The initial parameters considered to create the models have been identified starting from the characteristics of the materials (modulus of elasticity and specific weight) of buildings, according to Eurocodes. The updating models process has been conducted slightly varying the parameters and, therefore, the experimental tests for the characterization of materials were not performed. 

### 4.1. FEM for Structure A

In the complete three-dimensional (3D) model of Structure A, the reinforced concrete beams and columns are modeled as frame-type elements; the masonry infill panels, however, are modeled as shell type elements. The columns are assumed to be fixed at the base and diaphragm-type constrains are applied at each floor level. A linear analysis of the complete building frame with gravity loads was carried out to determine the critical parameters influencing the interaction of the key elements in the lower part with the rest of the building frame.

**Figure 18:**
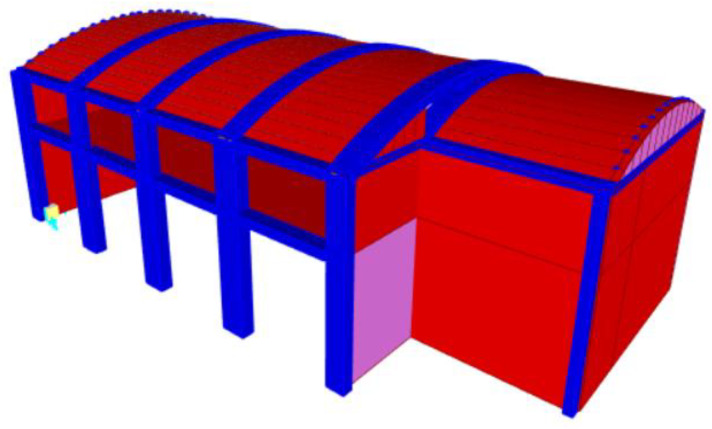
Three-dimensional (3D) Finite Element Model of the Structure A.

In particular, the material properties and the specific characteristics of the adopted FE models are reported in Table 3; in Figure 18, the 3D model is shown.

**Figure 19:**
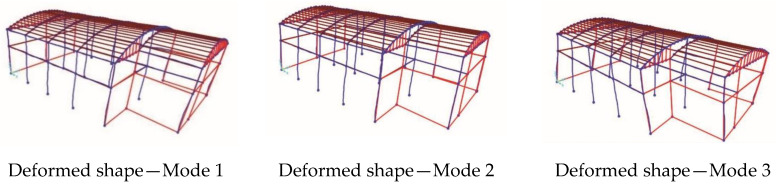
Modes of vibration of the FE model of Structure A.

The linear analysis (modal analysis) conducted by using FE model allowed to determine the deformed shapes corresponding to the first three vibration modes (Figure 19) and the numerical frequencies (f). In Table 4, the experimentally and analytically identified dynamic characteristics are compared for the first three modes.

In Table 4, the errors (in percentage) are indicated, they represent the gap between the average experimental frequency, and numerical frequency for each identified mode. It can be noted that such errors are very small, less than 5%.

### 4.2. FEM for Structure B

Structure B has been modeled with a complete three-dimensional (3D) frame, including reinforced concrete beams and columns as frame-type elements. The columns are assumed to be fixed at the base and the several floors are modeled by diaphragm type constrains applied at each floor level. Similar to Structure A, the linear analysis of the complete building frame subject to gravity loads was carried out to determine the critical parameters influencing the interaction of the key elements in the lower part with the rest of the building frame. 

**Figure 20:**
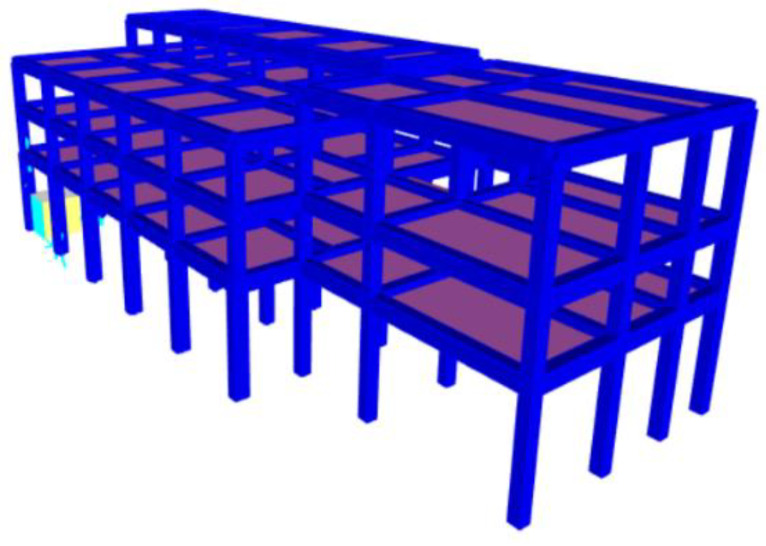
3D Finite Element Model of Structure B.

In particular, the material properties and the specific characteristics of the adopted FE model are detailed in Table 5; Figure 20 shows the 3D model.

**Figure 21:**
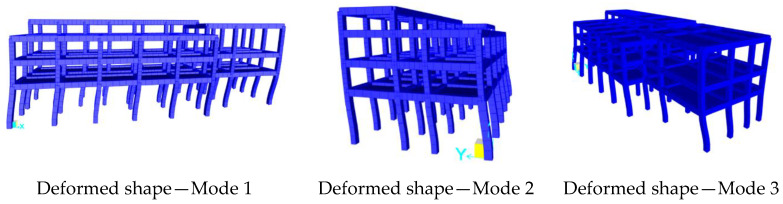
Modes of vibration of the FE model of Structure B.

In Figure 21, the deformed shape corresponding to the first three vibration modes of Structure B are reported. In Table 6, the experimentally and analytically identified dynamic characteristics (frequencies and mode types) are compared with each other for the first three modes. The difference between the average experimental frequency and numerical frequency is less than 9%.

## 5. Evaluation of Structural Operational Efficiency

The main purpose of the present paper is to demonstrate the utility of the direct use of validated FE models for evaluating the structural operativity of strategic buildings, fundamental for the management of emergencies. In fact, these buildings must not suffer damage such as to compromise their operation within a framework of assessment of the overall capacity of the urban system to satisfy the Emergency Limit Condition (ELC) [35]. Regarding the evaluation of this limit condition, the Seismic Model from Ambient Vibration (SMAV) methodology [26,36] has been applied to the structures analyzed and to their FE models. This methodology is based on the extraction of the experimental modal parameters of the structure, which are modal frequencies and mode shapes, used to calculate the seismic response of the structure through a dynamic linear analysis that operates by modal superposition. In addition, the methodology takes into account the decrease in natural frequencies, as deformation increases [37,38] through an iterative procedure based on three limit curves obtained from a probabilistic analysis, which expresses the decrease in natural frequencies in function of the maximum average drift (i.e., the maximum displacement of the last level with respect to the ground, divided by its height (H) with respect to the ground); it is named inter-plane drift. Finally, a Structural Operational Index (*IOPS*) for a given seismic action is proposed for the characterization of their vulnerability. The *IOPS* is determined as the ratio between the ultimate limit drift furnished by the code, which defines the Operating Limit State and the maximum drift obtained by SMAV when the building is subject to the reference earthquake.

The performance level required to meet the Emergency Limit Condition (ELC) is, therefore, the structural operation one, corresponding to the Operating Limit States (NTC2018 [15]). The performance level required by the ELC for the whole strategic building is that of structural operations organized by the Operating Limit State (OLS) envisaged by the NTC2018, specifically:

- Operational Limit State (OLS): following the earthquake, the construction as a whole, including the structural elements, the unprotected elements and the relevant equipment in relation to its function, must not suffer damage and interruptions of use (more stringent);
- Damage Limit State (DLS): following the earthquake, the construction as a whole, including the structural elements, the unstructured elements and the equipment relevant to its function, suffers damage from strength and stiffness against vertical and horizontal actions, remaining immediately usable despite interrupting the use of part of the equipment.

We have chosen to carry out the analysis for both limit states regarding the two structures under study, for which there are two different probabilities of overcoming the seismic action:

- Action 1 (OLS): probability of 10% in 50 years (reference is made to Class of use II and nominal life 50 years), corresponding to a return period of 475 years;
- Action 2 (DLS): probability of 63% in 100 years (reference is made to Class of use IV and nominal life 50 years), corresponding to a return period of 101 years.

**Figure 22:**
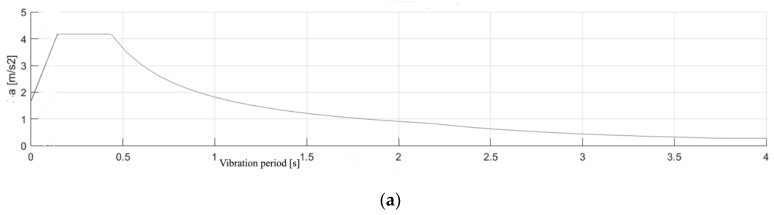

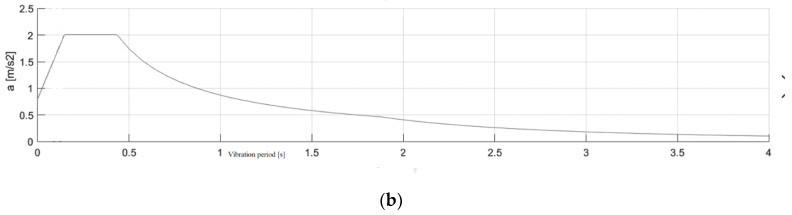
(**a**) Acceleration spectrum for seismic action 1; (**b**) acceleration spectrum for seismic action 2.

In both cases, opportune choices of subsoil and topography category were carried out for completing the analysis. Since the assessments are conducted in terms of interstory drifts and, therefore, in terms of displacements, elastic response spectra are used for both seismic actions. The seismic action is expressed without considering any structural factor, using the elastic response spectrum for both actions; the accelerograms corresponding to Action 1 and 2 are shown in Figure 22. 

The final evaluation of the building can be expressed through the *IOPS* for the two defined seismic action levels (Action 1 and 2). This index, *IOPS*, is given by the ratio between the plan drift threshold IDR (Interstory Drift Ratio), which marks the achievement of the structural damage condition (named *IDR_Limit_*) indicated by the NTC2018 and the maximum plan drift envisaged by the applied procedure (named *IDR_SMAV_*) as expressed in Equation (1).

(1) $$IOPS=\frac{IDR_{Limit}}{IDR_{SMAV}},$$

In relation to the seismic actions described, *IOPS*_475_ is calculated for Action 1, *IOPS*_100_ is calculated for Action 2; the check of the structural operation of the strategic buildings is considered positive if both the indicators *IOPS*_475_ and *IOPS*_100_ are greater than 1.

The challenge of the present manuscript is to demonstrate that the *IOPS* index about the structural operativity of two strategic and different buildings may be calculated also by considering their validated FE models, considering the FE models as virtual generators of the modal parameters necessary for the SMAV procedure. To this aim, the modal properties of the points of the model corresponding to the instrumented points of the structures have been considered as the virtual input of the procedure. It must be considered that the structures are very different from each other; only four points of measurement for Structure A and ten points of measurement for Structure B. Furthermore, their geometry and characteristics are completely different. Moreover, it must be considered that the validation of the FE models, for both the structures, has been carried out considering the frequencies values, which is a much simpler experimental task than reconstructing their corresponding mode shapes. 

### 5.1. Structure A

Structure A is analyzed with the SMAV methodology by introducing the points defining the first and second floors, the six points for each plane as shown in Figure 4 and considering it as a heavily buffered reinforced concrete building. Moreover, regarding the floors, a thickness of 30 cm and a density equal to 12 kN/m^3^ has been defined; for load bearing masonry, a thickness of 40 cm and a density equal to 22 kN/m^3^ have been considered. Finally, taking into account the mode shape residues for each considered frequency (the three identified frequencies), and the seismic inputs (spectrum in Figure 21), it is possible to calculate, through the assumed stiff behavior of each plane, the time histories of the accelerations and displacements in each of all the defined points of the structure (six points for the first floor, six points for the second floor, totally 12 points), the inter-floor drifts and, finally, the indices *IOPS*_475_ and *IOPS*_100_. 

#### 5.1.1. *IOPS* Calculation From the Experimental Data

For each experimental test (Test 1, Test 2, Test 3) carried out on Structure A, the modes residues along *x* and *y* directions have been extracted for each identified frequency in the four points monitored (A, B, C, D, in Figure 4). 

In Table 7, the modes residues related to Test 1 for the three identified frequencies (with SSI technique) for each monitored point/direction. The flexional behavior is evident along *x*-axis for the first frequency, considering the increase of the *x* residue along the vertical direction (the aligned points A-C, B-D), the flexional behavior along *y* for the second frequency, and the torsional behavior for the third frequency.

Finally, considering the mode shape residues for each frequency and the seismic inputs (spectrum in Figure 21), it is possible to calculate, through the assumed stiff behavior of each plane, the geometric thickness and the procedure of frequency shift [37,38] detailed by [26,36], the maximum accelerations and displacements along *x* and *y* directions in each of all the defined points of the structure (six points for the first floor, six points for the second floor, totally 12 points) and, consequently, the maximum interstory drifts and IDR for the different combinations of the seismic action along the *x* and *y* directions. 

In Table 8, the maximum displacements in the *x* and *y* directions for the 12 points (see Figure 4) defining the first and second floor of Structure A and in Table 9 the maximum interstory drifts for Test 1 with seismic action 1 (OLS) are shown. In Table 10 and Table 11 the maximum displacements and maximum interstory drifts with seismic action 2 (DLS) are shown. The monitored points A, B, C and D correspond to points 11, 13, 21, 23, respectively, while the points at the ground floor (from 1 to 6, Figure 4) are considered fixed or reference points. From the data carried out from Test 1, shown in Table 8 and Table 9 (under seismic action 1) and Table 10 and Table 11 (under seismic action 2), it is possible to extract the maximum displacement δ_max_ and the Maximum Interstory Drift % for each seismic action. In detail, for OLS (seismic action 1) δ_max_ = 2.69 mm (points 22 and 23) and the maximum drift % is 0.47, for DLS (seismic action 2) δ_max_ = 1.06 mm (points 22 and 23), and the maximum drift % is 0.18. 

Following the technical standards [15], the maximum drift % is compared with the fixed threshold of 3%. The first indicator given is the percentage of couple of points that do not overpass this limit, defining the first indicator operativity that, in this case, is 100% for both seismic actions, indicating that there is no possibility that the drift % could cross the threshold. Moreover, the technical standards [15] for use classes III and IV structures with load-bearing walls confined by structural elements in reinforced concrete, introduce a limit displacement *δ_LIM_* defined by Equation (2), where *h* is the maximum interstory expressed in mm, equal to 4500 mm for Structure A.

(2) $$\delta_{LIM}=\frac{2}{3}\cdot 0.0025\cdot h=7.5\;\mathrm{mm}$$

The maximum displacements *δ*_max_ for both the seismic actions should not be greater than *δ_LIM_* to satisfy the technical standard [15] conditions. The indicators *IOPS*_475_ and *IOPS*_100_ are defined, respectively, as the ratio between *δ_LIM_* and the maximum displacement *δ*_max_ for seismic action 1 and the ratio between *δ_LIM_* and the maximum displacement *δ*_max_ for seismic action 2. These indicators satisfy the technical standard conditions when they are greater than 1 and their values quantify the operativity of the structure after the seismic actions. From the data in Table 8 and Table 10 and from Equation (2) it is possible to calculate *IOPS*_475_ = 7.5/2.69 = 2.78 and *IOPS*_100_ = 7.5/1.06 = 7.07 from the data of Test 1.

Considering the modal data of the other experimental tests (Test 2 and Test 3), the maximum drift % and the indicators *IOPS*_475_ and *IOPS*_100_ for all the tests are reported in Table 12.

The results clearly show that the structural operativity of the strategic Structure A is fully demonstrated with the SMAV methodology (all indicators much greater than 1 and all the possible maximum ISD% lower than the limit value) applied by using the experimental identified modal parameters of the building. The experimental modal parameters extracted from different tests give very close values to each other for OLS as well as for DLS, demonstrating, once more, the repeatability of the conducted analysis.

#### 5.1.2. *IOPS* Calculation from the Model Data

The FE model of Structure A previously introduced has also been used for comparing the results of the SMAV procedure. The input of the procedure, apart from the structure geometry that is the same used for the *IOPS* calculation from experimental data, is given by the modal data (frequencies and modes shapes) extracted from the FE model. In particular, for the extraction of the mode shapes, the modal residues of the elements corresponding to the monitored points (A, B, C, and D) have been considered from the FE models. The results obtained for OLS and DLS are reported in Table 13. 

Comparing Table 12 and Table 13 it is noticed that the FE model gives structural operativity indicators information very close to the information obtained from the experimental mode shapes data, demonstrating the full operativity of Structure A. It is important to underline that the FE model validation has been carried out only considering the matching of the first three frequencies; so, a sufficiently tuned model may provide important and verified information about the structural operativity of strategic buildings. 

### 5.2. Structure B

In order to confirm the excellent results obtained, a more complex and important strategic building, Structure B, with five levels, a good regularity in height, but not good regularity in plan has been considered. In this case, since the center of the stiffness does not exactly coincide with the center of the masses at the different floor decks, the building assumes a slight torsional component also in correspondence with the translational modes of vibrating. The geometric description of the structure requested 57 points (indicated in Figure 10) and 11 points of measurements (a total of 22 accelerometers, as indicated in Figure 14).

Structure B is inserted in the SMAV procedure similarly to the previous building. In this case, five floors have been inserted as stiff planes with a total of 41 points necessary to describe the five floor decks from the first to fifth floor (extracted from the 57 points depicted in Figure 14 neglecting ten points at the ground floor and the inner points in the other five floors). Moreover, in regards to the floors, a thickness of 30 cm and a density equal to 20 kN/m^3^ has been considered for the first and the second floor, and a density of 17 kN/m^3^ for the upper floors, considering the geometry and that it is as a fragile buffered reinforced concrete building.

#### 5.2.1. *IOPS* Calculation From the Experimental Data

For each experimental test (Test 1, Test 2, Test 3) carried out on Structure B, the residues modes along the *x* and *y* directions have been extracted for each identified frequency in the 10 monitored points (A, B, C, D, E, G, H, I, L, M of Figure 10). 

**Figure 23:**
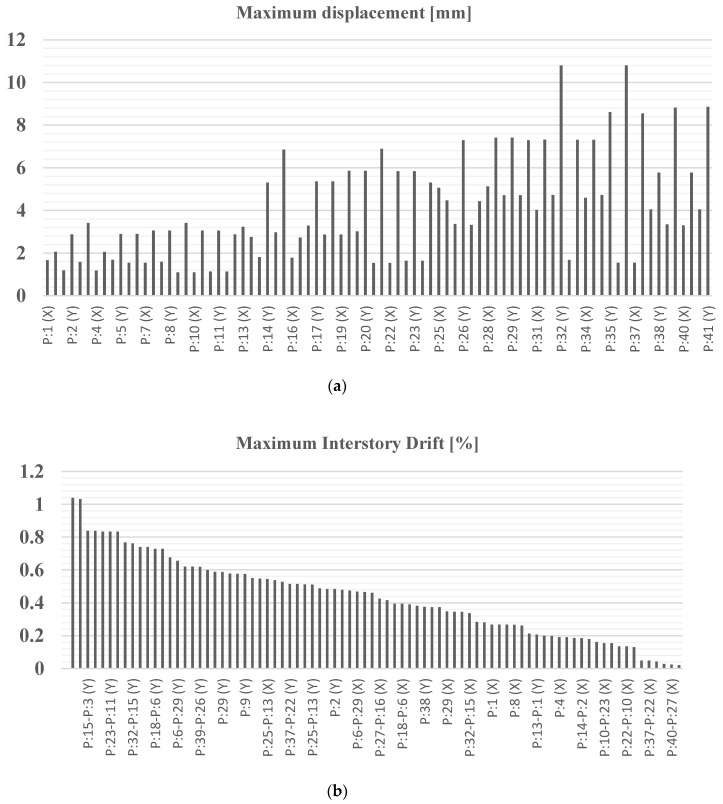
Structure B, Test 2, seismic action 2: (**a**) maximum displacement; (**b**) maximum interstory drift %.

Consequently, considering the mode shape residues for each considered frequency and the seismic inputs, similar for Structure A, it is possible to calculate the maximum accelerations and displacements along the *x* and *y* directions in each of the defined points of the structure (41 points, two directions for each points; therefore, totaling 82 values for the *x* and *y* components). Consequently, the maximum interstory drifts and IDR for the different combinations of seismic action on the *x* and *y* directions are determined. Figure 23 shows the maximum displacements in the *x* and *y* directions and the maximum interstory drift % for the 82 values of the 41 points defining the first, second, third, fourth and fifth floor of Structure B for Test 1 subject to a seismic action 1 (OLS) and a worst combination (100% *x*, 30% *y*). 

Considering the modal data of all the other experimental tests (Test 1, Test 2 and Test 3), and the limit displacement *δ_LIM_* calculated in (3) for strategic Structure B, with fragile infills rigidly connected to the supporting structure and a maximum height, h = 5.93 m, the maximum drift% and the indicators *IOPS*_475_ and *IOPS*_100_ for all the tests related to Structure B are reported in Table 13.

(3) $$\delta_{LIM}=\frac{2}{3}\cdot 0.005\cdot h=19.77\;\mathrm{mm}$$

The results in Table 14 show the structural operativity of the strategic Structure B; of course, the NTC2018 standards about the DLS tests are fully respected (ISD% is much lower than the threshold and for all the tests *IOPS*_100_ keeps sufficiently bigger than 1), giving a maximum probability of maintaining full operativity for seismic action 2. About OLS tests, the operativity of Structure B is not so fully reached. In fact, ISD % indicator is bigger than 3 for Test 1 and close to 2 for the other two tests, that is not so far from the threshold. In addition, *IOPS*_475_ varies from 0.76 and 0.81 (in Test 1 and 2), while it reaches the value of 1.49 for Test 3, that is just across the threshold equal to 1. 

The experimental modal parameters extracted from different tests show indicators that vary with the modal data of the tests considered. However, they give indicators close to the same type of operativity for OLS and for DLS too, demonstrating, once more, the repeatability of the conducted analysis.

#### 5.2.2. *IOPS* Calculation from the Model Data

A FE model previously introduced for Structure A has also been used for comparing the results of the SMAV procedure. Apart from the geometry of the structure that is the same used for the *IOPS* calculation from the experimental data, the input of the procedure is given by the modal data (frequencies and mode shapes) extracted from the FE model. In particular, for the extraction of the mode shapes, the modal residues of the elements corresponding to the monitored points (A, B, C, D, E, G, H, I, L, M) have been considered from the FE models. The results obtained for OLS and DLS are reported in Table 15. Comparing Table 14 with Table 15, it is evident that the FE model gives structural operativity indicators information very close to the information obtained from the experimental mode shapes data of Structure B. The *IOPS*_475_ obtained from the model data (1.38) is included between the corresponding values extracted from the experimental data (varying from 0.76 to 1.49 in the three tests). Similarly to the experimental results, the fully operativity obtained for DLS is confirmed also by the numerical model data. Moreover, *IOPS*_100_ obtained from the model data (3.05) is included in the range of *IOPS*_100_ obtained from the experimental data (from 1.83 to 3.3).

It is important to underline that, also in this more complex case, the FE model validation has been carried out only considering the matching of the first three frequencies; therefore, again, a sufficiently tuned model may provide important and verified information about the structural operativity of strategic buildings. 

## 6. Conclusions

This research has achieved the important objective of demonstrating that validated FE models of strategic buildings may be used for evaluating the structural operativity of these buildings in case of a seismic event. The validation used is based on the non-destructive identification, through environmental vibration measurements, of the dynamic characteristics of two buildings of strategic importance. The used dynamic identification techniques by environmental inputs belong to the "OMA" type and they use the installation on the structural elements of highly sensitive sensors able to acquire the vibrations of the structure itself. The great advantage of this technique is the possibility to operate without interrupting the activities inside the building during the monitoring activity. After the experimental evaluation of the dynamic characteristics of the structure, the structural operating index (*IOPS*) was calculated by means of the SMAV methodology, for the characterization of the operativity of these strategic buildings. In addition to the structural operating indicator, it has been possible to also extract the maximum floor displacements that could occur in the event of an earthquake. The seismic action has been simulated by two response spectra corresponding to OLS and DLS with the objective to evaluate the operativity of existing buildings. This means that the use of this methodology is limited to seismic forces that do not provoke significant nonlinearities in the behavior of the building. Therefore, SMAV is not able to investigate the building’s behavior at OLS and DLS limits. For this reason, in this paper it is proposed to systematically help the SMAV model with a calibrated finite element numerical model (with SMAV up to DLS) to evaluate the performance of the building at OLS and DLS.

The same SMAV methodology has also been applied to the data generated in a virtual way from the FE models of the buildings; the two models have been validated by comparing the first three identified frequencies with those experimentally obtained. 

It must be considered that the two strategic buildings analyzed are deeply different: the geometry (two floors the first, five floors the second), the infills, the results about the operativity (especially for OLS). In both cases, the results obtained from the virtual data are very close to the data obtained from the experimental data that, however, need the knowledge of the mode shapes related to the frequencies considered. Therefore, a much more complex experimental phase and a much bigger amount of instrumentation (accelerometers, acquisition boards, etc.) are required.

The advantages obtained from the analysis here developed are related to the speed of execution with the least invasiveness, the ease of implementation of the algorithm, the precision of the results, as they are based on the experimental data obtained by OMA, and the ability to model structurally complex buildings in a simple way. The methodology, at present, shows some critical issues related to the use of limit drift values, general frequency reduction curves, and to the essentially linear character of the SMAV model. However, in this paper, two applicative cases are shown, and the excellent results obtained make the authors aware that the proposed approach could have a wide range of applicability also for other buildings, if a well-tuned FE model is available.

In conclusion, this study allows us to understand the importance, for strategic buildings, of carrying out an in-depth study on their dynamic characteristics and the operational status. Thus, it will be possible to intervene promptly, since, in case of exceptional events, such as earthquakes, these buildings have a "strategic" role of control, monitoring, and intervention. Therefore, in such situations, it is not possible to interrupt the activity or, even worse, to suffer serious structural damage or collapses.

## Figures and Tables

**Table 1:** Experimental identified frequencies (Hz) by SSI and EFDD techniques and mode shape type.

STRUCTURE A.									
		TEST 1			TEST 2			TEST 3	
MODE	SSI	EFDD	Type	SSI	EFDD	Type	SSI	EFDD	Type
**1**	8.06	8.04	*x*_Flex	8.04	8.07	*x*_Flex	8.05	8.06	*x*_Flex
**2**	9.70	9.72	*y*_Flex	9.55	9.82	*y*_Flex	9.64	9.73	*y*_Flex
**3**	11.31	11.25	Tors	11.40	11.39	Tors	11.37	11.40	Tors

**Table 2:** Experimental identified frequencies [Hz] with SSI and EFDD techniques and mode shape type.

STRUCTURE B									
	TEST 1			TEST 2			TEST 3		
MODE	SSI	EFDD	Type	SSI	EFDD	Type	SSI	EFDD	Type
1	3.65	3.62	x_Flex	3.63	3.63	x_Flex	3.61	3.59	x_Flex
2	3.70	-	y_Flex	3.65	-	y_Flex	3.67	3.72	y_Flex
3	4.23	4.24	Tors	4.19	4.26	Tors	4.21	4.18	Tors

**Table 3:** Material properties and elements type adopted in the Finite Element (FE) models—Structure A.

	Material Properties			Element Type		
	Modulus of Elasticity (N/mm^2^)	Poisson’s Ratio (-)	Density (kN/m^3^)	Number of Nodes	Number of Frames	Number of Shells
**Reinforced Concrete**	31476	0.2	25	237	252	173
**Masonry**	1750	0.3	15.6			

**Table 4:** Numerical frequencies (f) and mode shape types obtained from the FE model—Structure A.

	FEM		OMA		
MODE	f(Hz)	Type	f (Hz)	Type	Error (%)
1	7.80	*x*_Flex	8.05	*x*_Flex	3.27
2	9.52	*y*_Flex	9.69	*y*_Flex	1.88
3	11.77	Torsional	11.35	Torsional	4.11

**Table 5:** Material properties and elements type adopted in the FE models—Structure B.

Material Properties				Element Type		
	Modulus of Elasticity (N/mm^2^)	Poisson’s Ratio (-)	Density (kN/m^3^)	Number of Nodes	Number of Frames	Number of Shells
**Reinforced Concrete**	36416	0.2	24	180	322	-

**Table 6:** Numerical frequencies (f) and mode shape types obtained from the FE model—Structure B.

	FEM		OMA		
MODE	f (Hz)	Type	f(Hz)	Type	Error (%)
1	3.53	*x*_Flex	3.62	*x*_Flex	2.82
2	3.62	*y*_Flex	3.69	*y*_Flex	0.35
3	3.85	Torsional	4.22	Torsional	8.57

**Table 7:** Experimental modes residues with SSI technique.

STRUCTURE A, TEST 1									
Point/Direction									
MODE	Freq. [Hz]	A/*x*	A/*y*	C/x	C/*y*	B/*x*	B/*y*	D/x	D/*y*
**1**	**8.06**	0.283	−0.021	0.591	−0.015	0.427	0.016	0.62	0.035
**2**	**9.70**	0.080	0.067	0.155	0.169	−0.124	0.533	−0.197	0.773
**3**	**11.31**	−0.125	0.347	−0.256	0.716	0.213	−0.237	0.323	−0.277

**Table 8:** Maximum displacements [mm] for each point, seismic action 1, combination (100% *x*, 30% *y*).

STRUCTURE A, TEST 1												
Direction	Points Maximum Displacements [mm]											
	11	12	13	14	15	16	21	22	23	24	25	26
*x*	1.57	1.53	1.53	1.56	1.56	1.58	2.60	2.69	2.69	2.62	2.62	2.60
*y*	0.34	0.24	0.34	0.24	0.20	0.20	0.55	0.46	0.55	0.46	0.40	0.40

**Table 9:** Maximum Interstory Drift [%], seismic action 1, combination (100% *x*, 30% y).

STRUCTURE A, TEST 1													
		Interstory Drifts [%]											
	Points	22-12	23-13	24-14	25-15	26-16	21-11	16-6	11-1	14-4	15-5	13-3	12-2
Direction													
*x*		0.47	0.47	0.43	0.43	0.42	0.42	0.35	0.35	0.35	0.35	0.34	0.34
*y*		0.09	0.11	0.09	0.08	0.08	0.11	0.04	0.07	0.05	0.04	0.07	0.05

**Table 10:** Maximum displacements [mm] for each point, seismic action 2, combination (100% *x*, 30% *y*).

STRUCTURE A, TEST 1												
Direction	Points Maximum Displacements [mm]											
	11	12	13	14	15	16	21	22	23	24	25	26
*x*	0.62	0.60	0.6	0.61	0.61	0.62	1.02	1.06	1.06	1.03	1.03	1.02
*y*	0.13	0.09	0.13	0.09	0.08	0.08	0.21	0.18	0.21	0.18	0.16	0.16

**Table 11:** Maximum interstory Drift [%], seismic action 2, combination (100% *x*, 30% *y*).

STRUCTURE A, TEST 1													
		Interstory Drifts [%]											
	Points	22-12	23-13	24-14	25-15	26-16	21-11	16-6	11-1	14-4	15-5	13-3	12-2
Direction													
*x*		0.18	0.18	0.17	0.17	0.16	0.16	0.14	0.14	0.14	0.14	0.13	0.13
*y*		0.03	0.04	0.04	0.03	0.03	0.04	0.02	0.03	0.02	0.02	0.03	0.02

**Table 12:** Maximum Interstory Drift (ISD) % and operativity indicators *IOPS*_475_ and *IOPS*_100_ for Operating Limit State (OLS) and Damage Limit State (DLS).

STRUCTURE A											
TEST 1				TEST 2				TEST 3			
OLS		DLS		OLS		DLS		OLS		DLS	
ISD%	IOP_S475_	ISD%	*IOPS*_100_	ISD%	*IOPS*_475_	ISD%	*IOPS*_100_	ISD%	*IOPS*_475_	ISD%	*IOPS*_100_
0.47	2.78	0.18	7.07	0.48	2.70	0.19	6.88	0.50	2.70	0.19	6.88

**Table 13:** Maximum Interstory Drift (ISD) % and operativity indicators *IOPS*475 and *IOPS*100 for OLS and DLS, respectively.

STRUCTURE A—FE MODEL			
OLS		DLS	
ISD%	*IOPS*_475_	ISD%	*IOPS*_100_
0.43	2.60	0.17	6.57

**Table 14:** Maximum Interstory Drift (ISD) % and operativity indicators *IOPS*_475_ and *IOPS*_100_ for OLS and DLS.

STRUCTURE B											
TEST 1				TEST 2				TEST 3			
OLS		DLS		OLS		DLS		OLS		DLS	
ISD%	*IOPS*_475_	ISD%	*IOPS*_100_	ISD%	*IOPS*_475_	ISD%	*IOPS*_100_	ISD%	*IOPS*_475_	ISD%	*IOPS*_100_
4.4	0.76	1.83	1.83	2.36	0.81	1.04	1.83	1.99	1.49	0.90	3.3

**Table 15:** Maximum Interstory Drift (ISD) % and operativity indicators *IOPS*475 and *IOPS*100 for OLS and DLS.

STRUCTURE B—FE MODEL			
OLS		DLS	
ISD %	*IOPS*_475_	ISD%	*IOPS*_100_
1.53	1.38	0.69	3.05
